# Evaluation of dietary *Bacillus*-based commercial probiotics in broiler chickens

**DOI:** 10.1093/jas/skag269

**Published:** 2026-08-26

**Authors:** Viet Anh Vu, Soo-Ki Kim, Huy Doanh Bui, Da-Hye Kim, Byung-Yeon Kwon, Jina Park, Ju-Yong Song, Gyu Bum Choi, Young Bin Park, Sung-Ho Lee, Kyung-Woo Lee

**Affiliations:** Department of Animal Science and Technology, Sanghuh College of Life Sciences, Konkuk University, Seoul, 05029, South Korea; Department of Animal Science and Technology, Sanghuh College of Life Sciences, Konkuk University, Seoul, 05029, South Korea; Department of Animal Biochemistry, Faculty of Animal Science, Vietnam National University of Agriculture, Ngo Xuan Quang, Hanoi, 12406, Vietnam; Department of Animal Biochemistry, Faculty of Animal Science, Vietnam National University of Agriculture, Ngo Xuan Quang, Hanoi, 12406, Vietnam; Department of Animal Science and Technology, Sanghuh College of Life Sciences, Konkuk University, Seoul, 05029, South Korea; Department of Animal Science and Technology, Sanghuh College of Life Sciences, Konkuk University, Seoul, 05029, South Korea; Department of Animal Science and Technology, Sanghuh College of Life Sciences, Konkuk University, Seoul, 05029, South Korea; Department of Animal Science and Technology, Sanghuh College of Life Sciences, Konkuk University, Seoul, 05029, South Korea; Department of Animal Science and Technology, Sanghuh College of Life Sciences, Konkuk University, Seoul, 05029, South Korea; Woogene B&G Co., Ltd., Gyeonggi-do, 18628, South Korea; Woogene B&G Co., Ltd., Gyeonggi-do, 18628, South Korea; Department of Animal Science and Technology, Sanghuh College of Life Sciences, Konkuk University, Seoul, 05029, South Korea

**Keywords:** broiler, *Bacillus* spp. probiotic, growth performance, gut health, immunity

## Abstract

Commercial *Bacillus*-based probiotics are widely used in poultry production, but their biological mechanisms of action in broiler chickens are still not fully understood. This study evaluated the effects of dietary supplementation with *Bacillus*-based commercial probiotics on the performance, meat and tibia traits, gut health (ileal morphology and cecal volatile fatty acids), antioxidant status, nutrient digestibility, and intestinal gene expression in broiler chickens. Day-old Ross 308 broiler chicks (288 in total) were randomly assigned to one of four dietary treatments, with six replicate pens of 12 birds each in an environmentally controlled facility. The treatments included a control group fed a basal diet and three probiotic-supplemented diets: *Bacillus subtilis* KCCM13366P at 5.0 × 10^7^ cfu/kg (BS), *Bacillus licheniformis* KCCM11751P at 1.0 × 10^8^ cfu/kg (BL), or an equal blend at 2.0 × 10^8^ cfu/kg (BSL). On days 21 and 35, one or three birds per pen were sampled for feathers, blood, small intestine, and cecal digesta to determine the indicators of gut health (i.e. secrtory immunoglobulin A, gut morphology, cecal volatile fatty acids, and intestinal gene expression) and physiology (i.e. serum parameters, corticosterone, and antioxidants parameters). In addition, meat quality and ileal nutrient digestibility were assayed on day 35. Data were analyzed using the general linear model procedure in SAS followed by pairwise differences using Tukey’s post-hoc test. Statistical significance was preset at *P *< 0.05. The trial lasted for 35 days. Dietary BSL tended to increase feed intake during the grower phase by 6.33% (*P *= 0.087). Dietary BS and BSL improved the feed conversion ratio by 7.83% and 5.22%, respectively, during the starter phase (*P *< 0.05) and BSL reduced overall feed conversion ratio by 1.99% compared to the control group (*P *< 0.05). At 21 days, dietary BSL increased the ileal villus height: crypt depth ratio by 40.66% (*P *< 0.05). The activity of glutathione peroxidase in the ileal mucosa, but not in the serum samples, was decreased by 36.34% in the BS group and 38.06% in the BSL group at 21 days (*P *< 0.05). Serum glutamic oxaloacetic transaminase concentrations were lower by 22.3% on day 21, and ileal digestibility of crude protein and crude ash were lower by 6.6% and 21.9% on day 35, respectively, in birds fed the BSL diet than in the control group (*P *< 0.05). The interleukin-8 and interferon-alpha expression levels in the ileum were increased by 2.0-fold in BL-fed broilers compared to the control group (*P *< 0.05). The expression of genes encoding antioxidant-related parameters, tight junction proteins, mucin, and glucose transporters was upregulated by 2.0- to 4.0-fold in dietary BL and BSL groups on days 21 and 35 (*P *< 0.05). We concluded that dietary BS improved growth performance, whereas dietary BL enhanced cytokine, gut barrier, mucin, antioxidant, and nutrient transporter mRNA levels, resulting in additive effects of BSL on chickens. Based on the enhanced intestinal gene expression induced by dietary BS or BSL, further studies are needed to determine protective immunity against enteric pathogens in broiler chickens.

## Teaser

Although *Bacillus*-based probiotics have been shown to improve growth performance in chickens, their underlying cellular and molecular mechanisms remain unclear. This study provides new insight into the bioefficacy of commercial *Bacillus*-based preparations by linking growth responses with intestinal gene expression related to immune regulation, barrier function, and nutrient transport.

## Introduction

Probiotics are defined as “live microorganisms that, when administered in adequate amounts, confer a health benefit on the host” and have emerged as a primary strategy for reinforcing the intestinal barrier and optimizing gut health in chickens (Jha et al. 2020). Among the diverse array of probiotic candidates, spore-forming bacteria belonging to the genus *Bacillus* have attracted particular attention owing to their industrial suitability (Elshaghabee et al. 2017). *Bacillus* spp. naturally form metabolically dormant endosporesabx that possess exceptional resistance to environmental stress (Elshaghabee et al. 2017). This biological resilience enables them to survive the feed manufacturing process (Ramlucken et al. 2020b) and acidic gastric microenviroment of the avian proventriculus (Ramirez-Olea et al. 2022), ensuring the delivery of viable metabolic agents to the hindgut. *B. subtilis* and *B. licheniformis* are the most prominent species utilized in commercial formulations (Khalid et al. 2022). Their proposed modes of action include competitive exclusion of pathogens via oxygen consumption, synthesis of antimicrobial peptides, and secretion of exogenous enzymes that may complement the host’s digestive capacity (Ramlucken et al. 2020a).

Commercially available *Bacillus*-based probiotics have been marketed as single-strain (Mushtaq et al. 2024; Chen et al. 2025), multi-strain (Ramlucken et al. 2021; Horyanto et al. 2024), or multi-species (Neveling et al. 2020; Reuben et al. 2021). In addition, the inclusion levels of *Bacillus*-based probiotics in broilers ranged from as low as 1 × 10^5^ cfu/kg diet to as high as 1 × 10^10^ cfu/kg diet (Zhang et al. 2013; Bai et al. 2017; Upadhaya et al. 2019; Kan et al. 2021; Liu et al. 2021), indicating that the optimal dosages for their effectiveness might be strain- or species-dependent. Although numerous studies have documented phenotypic improvements in the average daily gain and feed conversion ratio in chickens (Liu et al. 2012; Xu et al. 2021), the underlying cellular and molecular mechanisms of *Bacillus*-based probiotic preparations remain unclear. In addition, the effects of these probiotics on the physical integrity of the intestinal barrier, antioxidant defense, and nutrient transport capacities remain largely underexplored. Therefore, the aim of this study was to evaluate three commercial *Bacillus*-based products: *B. subtilis*, *B. licheniformis*, and their combination on growth performance, meat and bone quality, ileal morphology, stress indicators, serum biochemical indices, antioxidant capacity, short-chain fatty acids, and the expression of genes associated with immune regulation, barrier integrity, and nutrient transport in broiler chickens to delineate the bioefficacy and underlying mechanisms of each probiotic formulation.

## Materials and methods

### Animal care

All experimental protocols were carefully examined and approved by the Institutional Animal Care and Use Committee of Konkuk University (KU 23212).

### Source of probiotics

The current study used probiotic preparations commercially manufactured by Woogene B&G (Seoul, Republic of Korea). These commercial preparations consisted of spore-forming bacteria with three distinct formulations: *B. subtilis* KCCM13366P at 1.0 × 10^8^ cfu/g (Sporezyme-forte, Woogene B&G Co., Hwaseong-si, Gyeonggi-do, South Korea), *B. licheniformis* KCCM11751P at 1.0 × 10^8^ cfu/g (Syner-win, Woogene B&G Co., Hwaseong-si, Gyeonggi-do, South Korea), and an equal blend of *Bacillus subtilis* KCCM13366P at 1.0 × 10^8^ cfu/g and *Bacillus licheniformis* at KCCM11751P 1.0 × 10^8^ cfu/g (Syner-win Plus, Woogene B&G Co., Hwaseong-si, Gyeonggi-do, South Korea). The inclusion levels of *Bacillus*-based probiotic preparations were as recommended by the manufacturer.

### Animals, diets, and experimental design

Day-old broiler chicks (Ross 308; 288 in total) were obtained from a local hatchery. Upon arrival, the chicks were individually weighed and randomly assigned to one of four dietary treatments, with six replicates of 12 birds each. Each pen (2 m × 1 m) contained fresh rice hulls as bedding material. Initially, the chicken facility was maintained at 32 °C, and the temperature was gradually decreased to 24 °C over 3 weeks, after which it remained constant. All chicks had ad libitum access to food and water and maintained under a 23 h light:1 h dark photoperiod throughout the experimental period. The feeding trial lasted 5 weeks.

The basal diet was formulated using corn and soybean meal (Table 1). The experimental diets were formulated by mixing the control diet with *Bacillus*-based probiotic preparations to reach *B. subtilis* at 5.0 × 10^7^ cfu/kg (BS), *B. licheniformis* at 1.0 × 10^8^ cfu/kg (BL), and the equal blend of BS and BL at 2.0 × 10^8^ cfu/kg (BSL). Bird-related mortalities were recorded daily during the study period. On days 0, 21, and 35, the feed intake and body weight of each replicate were recorded. The feed intake, body weight gain, and mortality-adjusted feed conversion ratios were calculated.

**Table 1:** Ingredients and chemical composition of the basal diet (%, as-fed).^1^

	g/100 g
**Ingredients**	
**Corn**	56.66
**Soybean meal, 44.7% CP**	29.00
**Corn gluten meal**	7.00
**Animal fat**	2.00
**Iodized salt**	0.30
**Monocalcium phosphate**	1.30
**_DL_-Methionine, 99%**	0.35
**_L_-Lysine-HCl, 56%**	0.50
**_L_-Threonine, 99%**	0.10
**Ground limestone**	1.90
**Sodium bicarbonate**	0.24
**Choline chloride, 50%**	0.20
**Vitamin premix^2^**	0.20
**Mineral premix^3^**	0.25
**Total**	100.00
**Calculated nutrient composition, %**	
**AMEn, kcal/kg**	3,039
**Dry matter^4^**	88.8
**Crude protein^4^**	24.8
**Calcium**	1.02
**Total phosphorus**	0.71
**Available phosphorus**	0.45
**Chloride**	0.21
**Sodium**	0.21
**Lysine**	1.33
**Methionine**	0.72
**Methionine + cysteine**	1.08
**Threonine**	0.92
**Arginine**	1.29
**Histidine**	0.55

1 CP, crude protein; AMEn, nitrogen-corrected apparent metabolizable energy.

2 Vitamin premix provided the following nutrients per kg of diet: vitamin A, 9,000 IU; vitamin D_3_, 4,000 IU; vitamin E, 58 mg; vitamin K_3_, 2.7 mg; thiamine, 2.3 mg; riboflavin, 5.9 mg; vitamin B_5_, 17 mg; vitamin B_6_, 2.9 mg; vitamin B_12_, 0.015 mg; niacin, 54 mg; biotin, 0.16 mg; folate, 1.7 mg.

3 Mineral premix provided the following nutrients per kg of diet: Fe, 57.1 mg; Mn, 85.7 mg; Zn, 64.3 mg; I, 0.57 mg; Se, 0.2 mg; Cu, 100 mg; Co, 0.17 mg.

4 Analyzed values.

### Sample collection

On days 21 and 35, feathers from the interscapular area were collected from 3 birds per pen. In addition, one bird per pen was selected and euthanized using an overdose of carbon dioxide. Immediately after euthanasia, blood was collected via heart puncture and serum samples were separated via centrifugation at 200 × *g* for 15 min and stored at −20 °C until use. After blood sampling, the ileal segment and the pair of ceca were sampled. Ileal segments were excised from 2 cm posterior to the Meckel’s diverticulum to 2 cm anterior to the ileocecal valve. Then, a 1 cm mid-ileal segment was excised, washed, and stored in 10% neutral-buffered formalin for ileal morphology. The first half of the ileal segment (to assay secretory immunoglobulin A; sIgA) was cut longitudinally and rinsed with PBS to remove the luminal contents. Then, mucosal samples were collected by gently scraping the ileal surface using a tissue-culture scraper, homogenized, centrifuged at 1,000 × *g* at 4 °C for 10 min, and stored at −20 °C until use. The terminal ileum (intended for assay gene expression analysis) was dissected, thoroughly rinsed to remove residual luminal contents, and immediately placed in RNAlater (Invitrogen, Carlsbad, CA, USA). A pair of ceca was kept on ice and processed to determine the short-chain fatty acid concentrations on the day of sampling. In addition to the samples described above, right breast and leg meat samples were collected on day 35 and expressed relative to live body weight. The tibia was collected by carefully excising the surrounding muscle, meat, and connective tissue and weighing.

All birds were fed diets containing 2% celite as an indigestible marker between days 28 and 35 to assess the apparent ileal digestibility (AID) of nutrients. On day 35, five birds per pen were randomly selected and humanely euthanized using carbon dioxide. Immediately after euthanasia, ileal digesta were carefully collected by gently stripping the ileal segment, pooled per pen, and stored at –20 °C until use.

### Measurements of meat quality

On day 35, one bird per replicate was sampled for the breast and leg muscles to assess cooking loss, pH, and color. To determine the water holding capacity, breast and deboned leg meat samples were vacuum-sealed in plastic bags and submerged in an 80 °C water bath for 30 min. After cooking, the samples were rapidly cooled in cold water on ice for 20 min, as previously described (Vu et al. 2025). Tissue paper was used to absorb the residual moisture from the meat surface. The difference in weight between raw and cooked meat was used to evaluate cooking loss. The pH values of breast and leg meat were measured at three different points using a portable pH meter (Testo 205, AG, Germany). The color of fresh meat, including lightness (L*), redness (a*), and yellowness (b*), was evaluated using a reflectance colorimeter (CM-2600d/2500d; Minolta, Japan). Measurements were performed three times per sample, and the colorimeter was calibrated using a standard white ceramic tile.

### Measurements of tibia characteristics

The width and length of the tibia were measured with Vernier calipers. The ability of the tibia to withstand breaking was assessed using an Instron Universal Testing Machine (Model 3342, Instron Co., Norwood, MA, USA), operating at a 50-kg load and 50 mm/min crosshead speed, with the bone resting on a 3.35 cm span.

### Measurements of ileal morphology

Morphological sections (4-μm thickness) were mounted on slides and stained with hematoxylin-eosin solution. Villi height and crypt depth were measured in ten intact villi and crypts under a digital microscope at 40× magnification (Olympus, Tokyo, Japan), and images were recorded using a digital camera (eXcope T500, DIXI Science, Daejeon, Republic of Korea). Villus height was measured from the top of the villus to its bottom, and crypt depth was defined as the length from the base of the villus to the crypt. The ratio of villus height to crypt depth was also calculated.

### Measurements of corticosterone in serum and feather samples

Corticosterone levels were assayed to determine whether dietary probiotic preparations could mitigate stress responses in broilers. Corticosterone in feathers sampled on days 21 and 35 was extracted using the methanol-based extraction protocol described by Kwon et al. (2024). Serum and extracted feather samples were analyzed using a corticosterone ELISA kit (ADI-901-097, Enzo Life Science Inc., Farmingdale, NY, USA) following the manufacturer’s recommendations.

### Measurements of antioxidant parameters in serum and ileal mucosa samples

Serum and ileal mucosa samples were analyzed for glutathione peroxidase (GPX) using the EnzyChrom assay kit (BioAssay Systems, Hayward, CA, USA), superoxide dismutase (SOD) activity using the EnzyChrom assay kit (BioAssay Systems), total antioxidant capacity (TAC) using the QuantiChrom assay kit (BioAssay Systems), catalase (CAT) activity using the OxiSelect assay kit, and malondialdehyde (MDA) using the OxiSelect thiobarbituric acid reactive substances assay kit (Cell Biolabs, Inc, San Diego, CA, USA). The protein concentration in ileal mucosa samples was determined using Bradford reagent (Sigma-Aldrich, St. Louis, MO, USA).

### Measurement of sIgA contents in ileal mucosa

The sIgA levels in the mucosal homogenates were assessed using commercially available ELISA kits (Bethyl Laboratories, Montgomery, TX, USA) following the manufacturer’s instructions. The protein concentration in ileal mucosa samples was determined using Bradford reagent (Sigma-Aldrich). sIgA contents were expressed as μg sIgA per mg of total protein.

### Measurements of short-chain fatty acids in cecal samples

Approximately 1 g of cecal digesta was combined with 4 mL of distilled water, thoroughly mixed using a vortex mixer (C-VT Test Tube Voltex Mixer, Chang Shin Scientific Co, Incheon, KOREA), and added with 0.05 mL of saturated HgCl_2_, 1 mL of 25% H_3_PO_4_, and 0.2 mL of 2% pivalic acid followed by centrifugation at 1,000 × *g* and 4 °C for 20 min. The supernatant was then used to quantify the concentrations of short-chain fatty acids in the cecal digesta samples by gas chromatography (6890 Series GC System, Hewlett Packard, Palo Alto, CA, USA) per the methodology described by Lee et al. (2022).

### Measurements of serum biochemical profiles and nitric oxide

Serum biochemical parameters, including total cholesterol, triglycerides, high-density lipoprotein cholesterol, total protein, albumin, globulin, glucose, glutamic pyruvic transaminase (GPT), glutamic oxaloacetic transaminase (GOT), uric acid, and creatinine, were quantified using an automated clinical chemistry analyzer (Film DRI CHEM 7000i, Fuji Film Co., Ltd., Tokyo, Japan). In addition, nitric oxide concentrations in serum samples were determined as described previously (Ndazigaruye et al. 2019).

### Nutrient digestibility

Both feed and ileal digesta samples were analyzed (AOAC 2019) for dry matter (method 930.15), crude protein (method 990.03), ether extract (method 920.39), ash (method 942.05), and neutral detergent fiber (Van Soest et al. 1991). Celite, which was used as an inert marker, was analyzed for acid-insoluble ash (AIA) following the procedure described by Dourado et al. (2010). The AID of the nutrients was calculated using the following equation:

AID = 100 − [(feed indicator %/ileal indicator %) × (ileal nutrient %/feed nutrient %) × 100]

### RNA extraction, cDNA synthesis, and real-time PCR analysis

Approximately 50 mg of ileal tissue was homogenized in 1 mL of RiboEx reagent using a homogenizer (GeneAll, Hanam, Gyeonggi, ROKA). Total RNA was isolated from the ileum using RiboEx reagent following the manufacturer’s protocol. The isolated RNA was standardized to a uniform concentration (1000 ng) for all samples and converted into cDNA using a RevertAid First Strand cDNA Synthesis Kit (Molecular Biology, Thermo Fisher Scientific, Waltham, MA, USA). National Center for Biotechnology Information (NCBI) was used to design primers for genes involved in gut barriers such as claudin (CLDN)-2, CLDN1, junctional adhesion molecule-2 (JAM2), occluding (OCLDN), zonula occludens (ZO)-1, ZO-2, and mucin (MUC)-2, inflammation-related factors including factor nuclear factor kappa B (NF-kB) and toll-like receptor 4 (TLR-4), inflammatory cytokines including interleukin (IL)-6, IL-8, IL-12, IL-2, IL-18, IL-1β, IL-10, IL-4, interferon-α (IFN-α), tumor necrosis factor alpha (TNF-α), and tumor necrosis factor superfamily, member 15 (TNFSF-15), antioxidant-related genes including glutathione peroxidase (GPx)-1 and superoxide dismutase (SOD)-1, and glucose transporter (GLUT)-2. For real-time PCR, cDNA samples were combined with Power SYBR Green PCR Master Mix (Applied BioSystems, Thermo Fisher Scientific, Waltham, MA, USA) and analyzed using the QuantStudio system (Applied BioSystems, Thermo Fisher Scientific). Glyceraldehyde-3-phosphate dehydrogenase (GAPDH) was used as an internal control to normalize target gene expression. The 2^–ΔΔCt^ approach was used to evaluate the expression of target genes relative to a reference housekeeping gene. The primers used in the RT-qPCR assay for the amplification of cytokine-related genes are presented in Table 2. Each sample was subjected to real-time PCR in triplicate.

**Table 2:** Primers used in real-time PCR.

Target gene	Primer sequence (5′-3′)*	Accession No.
**GAPDH**	F: TCTCTGTTGTTGACCTGACCTG R: CAACCTGGTCCTCTGTGTATCC	NM_204305.2
**IL-1β**	F: CTGACCCGCTTCATCTTCTACC R: TTAGCTTGTAGGTGGCGATGTT	NM_204524.2
**IL-2**	F: CAAACTCTGCAGTGTTACCTGG R: TAGACCCGTAAGACTCTTGAGG	NM_204153.2
**IL-4**	F: GGAGATAACAACCTAGACCAAACTG R: GATATCCTCCAAGCGCTTCCAT	NM_001007079.2
**IL-6**	F: AACTATCTATGAAGGCCGCTCC R: TCTGTCACACGGTAACAACACA	NM_204628.2
**IL-8**	F: GCTCAATTCTGATGCACCACTG R: TCATGATGCCTTCTTCGGATGT	HM179639.1
**IL-10**	F: CGTTCGGTCCGCTATTTAACAG R: TACAACAGCACTGCCACAAATG	NM_001004414.4
**IL-12**	F: CAGCGCATCCAACTTCTAAGC R: GTTTCGGGAGCTCACAATTGAA	NM_001398447.1
**IL-18**	F: TCAGCAGCTTACTCATTCCGTT R: TCAGCATTCTCGTCTTCAGCTT	NM_204608.3
**IFN- α**	F: CTCCAAGACAACGATTACAGC R: AAATAGGACAAGGGTGGGAG	NM_205427.1
**TNF- α**	F: TTCCTGCTGGGGTGCATAG R: AAGAACCAACGTGGGCATT	NM_204267
**TNFSF15**	F: CTGAGTTATTCCAGCAACGCA R: ATCCACCAGCTTGATGTCACT	NM_001024578
**NF-kB**	F: GAAGGAATCGTACCGGGAACA R: CTCAGAGGGCCTTGTGACAGTAA	NM_205134
**TLR4**	F: GAACATCTCTGGAGTTCCTGCT R: GCACCTTGAAAGATCCAGAAAC	NM_001030693.2
**CLDN1**	F: GTGTTCAGAGGCATCAGGTATC R: TCAGGTCAAACAGAGGTACAA	NM_001013611.2
**CLDN2**	F: CCTGCTCACCCTCATTGGAG R: GCTGAACTCACTCTTGGGCT	NM_001277622.1
**OCLDN**	F: GCAGAGGAGTACAATCAGCTCA R: GCTTGATGTGGAAGAGCTTGTT	NM_205128.1
**JAM2**	F: AATTTACAGTTCCTCCCACT R: TTCTTGTGGCAGTGCTGGCT	NM_001006257.1
**ZO1**	F: GAGTACGAGCAGTCAACATAC R: AGGCGCACGATCTTCATAA	XM_413773
**ZO2**	F: AGAAGCGTTGACAGGGATG R: GAAGGACTACGAGAATGAAGCC	NM_204918.1
**MUC2**	F: TTCATGATGCCTGCTCTTGTG R: CCTGAGCCTTGGTACATTCTTGT	XM_421035.2
**SOD-1**	F: CTCTGTAGTCTTCTGCTAAAGC R: CTGGAGCTCCTAAGACTCCTCA	NM_205064.2
**GPx-1**	F: CACCTCTGTCATACAAAGAGCT R: CATTTCTCCACGCAGCTTTGAA	NM_001277853.3
**GLUT2**	F: GCATTCCGCCGCAAGAA R: TTCCTTCTGCATCCCTCC	NM_207178.2

*F, forward; R, reverse.

### Statistical analysis

The sample size was determined by an a priori power analysis using a significance level (α) of 0.05, an expected treatment difference of 13%, and a coefficient of variation (CV) of 7.5%, which represents the expected experimental variability. Based on these assumptions, six replicates per treatment and 12 birds per replicate were considered sufficient. Similar sample sizes have also been used in previous studies (Vimon et al. 2020, 2023). Data were analyzed using the GLM procedure in SAS (SAS 9.4, SAS Inst. Inc., Cary, NC, USA). The model included the dietary treatment as a fixed variable. The least squares means were calculated for each treatment using Tukey’s post-hoc test to compare pairwise differences. Statistical significance was preset at *P *< 0.05 and the trend was defined at 0.05 ≤ *P *≤ 0.10.

## Results

### Effect on growth performance

None of the commercial dietary probiotics affected the body weight or body weight gain at any age (*P *> 0.05). The dietary treatments did not alter feed intake during the starter and overall phases (*P *> 0.05). However, dietary BSL, but not BS or BL, tended to increase feed intake by 6.33% compared with the control group during the grower phase (*P *= 0.087). Both dietary BS and BSL lowered the feed conversion ratio during the starter phase by 7.83% and 5.22%, respectively, and BSL reduced the overall feed conversion ratio by 1.99% compared to the control group (*P *< 0.05; Table 3).

**Table 3:** Effects of dietary *Bacillus*-based commercial probiotics on growth performance in broiler chickens.^1^

Item^2^	Treatments^3^				SEM^4^	*P*-value
	CON	BS	BL	BSL		
**BW, g/bird**						
**Day 0**	42.59	42.53	42.24	42.41	0.131	0.367
**Day 21**	790.0	829.8	808.0	835.8	14.40	0.129
**Day 35**	2042.5	2077.7	2035.0	2118.2	35.80	0.360
**BWG, g/bird**						
**Day 0 to 21**	747.3	787.3	765.7	793.2	14.36	0.127
**Day 21 to 35**	1252.5	1224.4	1227.2	1282.3	30.06	0.523
**Day 0 to 35**	1999.8	2035.0	1993.0	2075.5	35.80	0.365
**FI, g/bird**						
**Day 0 to 21**	1115.8	1093.7	1088.0	1122.5	20.49	0.582
**Day 21 to 35**	1793.7	1822.3	1807.7	1907.4	30.01	0.087
**Day 0 to 35**	2911.9	2912.9	2893.5	2976.7	52.17	0.695
**FCR, g:g**						
**Day 0 to 21**	1.494^a^	1.377^b^	1.420^a,b^	1.416^b^	0.019	0.004
**Day 21 to 35**	1.433	1.462	1.462	1.447	0.015	0.507
**Day 0 to 35**	1.456^a^	1.427^a,b^	1.452^a,b^	1.427^b^	0.007	0.014

1 Values are the least squares means representing six observations.

2 BW, body weight; BWG, body weight gain; FI, feed intake; FCR, feed conversion ratio.

3 CON, control diet; BS, control + Sporezyme-forte (*Bacillus subtilis* 5.0 × 10^7^ cfu/kg diet); BL, control + Syner-win (*Bacillus licheniformis* 1.0 × 10^8^ cfu/kg diet); BSL, control + Syner-win Plus (*Bacillus subtilis* and *Bacillus licheniformis* each 1.0 × 10^8^ cfu/kg diet, total 2.0 × 10^8^ cfu/kg diet).

4 SEM, standard errors of the means.

a,b Values with different superscripts within a row differ significantly (*P *< 0.05).

### Effect on meat quality and tibia traits at 35 days

None of the dietary probiotics affected the relative yield and quality, such as cooking loss; CIE L*, a*, and b* in breast and leg meats, or tibia traits, including fresh weight, breaking strength, length, and width (*P *> 0.05; Table 4). However, dietary BS significantly lowered breast meat pH by 2.21% compared to the control group (*P *< 0.05).

**Table 4:** Effects of dietary *Bacillus*-based commercial probiotics on meat and tibia traits in broiler chickens.^1^

Item	Treatments				SEM^3^	*P*-value
	CON^2^	BS	BL	BSL		
**Day 35**						
**Breast**						
**Meat yield, g per 100 g body weight**	11.84	12.31	12.42	13.20	0.673	0.561
**Cooking loss, %**	16.39	14.39	13.91	14.42	0.991	0.346
CIE L* (lightness)	51.72	52.43	51.23	51.30	1.183	0.854
CIE a* (redness)	1.753	1.047	1.794	1.462	0.382	0.518
CIE b* (yellowness)	14.36	14.49	14.44	13.85	0.631	0.899
**pH**	5.985^a^	5.853^b^	5.945^a,b^	5.990^a^	0.029	0.012
**Leg**						
**Meat yield, g per 100 g body weight**	8.407	8.540	8.429	8.893	0.377	0.786
**Cooking loss, %**	22.01	21.12	21.18	20.45	1.352	0.880
CIE L* (lightness)	53.75	54.24	53.73	52.48	0.699	0.220
CIE a* (redness)	6.292	6.288	5.515	6.415	0.464	0.441
CIE b* (yellowness)	17.52	17.01	16.97	17.17	0.755	0.953
**pH**	6.104	6.113	6.128	6.263	0.063	0.274
**Tibia traits**						
**Fresh weight, g/100 g of live BW**	0.930	0.867	0.921	1.010	0.038	0.094
**Breaking strength, kgf**	40.88	40.61	41.72	44.44	2.968	0.791
**Length, mm**	106.6	108.5	109.7	109.8	1.000	0.126
**Width, mm**	8.347	8.190	8.753	9.163	0.330	0.190

1 Values are the least squares means representing six observations.

2 CON, control diet; BS, control + Sporezyme-forte (*Bacillus subtilis* 5.0 × 10^7^ cfu/kg diet); BL, control + Syner-win (*Bacillus licheniformis* 1.0 × 10^8^ cfu/kg diet); BSL, control + Syner-win Plus (*Bacillus subtilis* and *Bacillus licheniformis* each 1.0 × 10^8^ cfu/kg diet, total 2.0 × 10^8^ cfu/kg diet).

3 SEM, standard errors of the means.

a,b Values with different superscripts within a row differ significantly (*P *< 0.05).

### Effect on ileal morphology

On day 21, villus height was not altered by dietary treatment (*P *> 0.05; Table 5). Dietary BSL, but not BS or BL, significantly decreased crypt depth by 17.52%, but increased villus height: crypt depth ratio by 40.66% compared with the control group (*P *< 0.05). On day 35, dietary probiotics did not affect villus height or villus height: crypt depth ratio compared with the control group (*P *> 0.05). Although crypt depth was lowest in the BL group and highest in the BSL group, statistical significance was not observed when compared with the control group.

**Table 5:** Effects of dietary *Bacillus*-based commercial probiotics on ileal morphology in broiler chickens.^1^

Item^2^	Treatments				SEM^4^	*P*-value
	CON^3^	BS	BL	BSL		
**Day 21**						
**Villus height, µm**	749.1	832.3	908.0	861.6	40.51	0.106
**Crypt depth, µm**	113.4^a^	104.4^a,b^	112.7^a^	93.53^b^	4.876	0.041
**VH to CD ratio**	6.633^b^	8.067^a,b^	7.401^a,b^	9.330^a^	0.556	0.023
**Day 35**						
**Villus height, µm**	804.1	825.3	846.1	896.1	44.05	0.556
**Crypt depth, µm**	103.0^a,b^	100.0^a,b^	87.38^b^	107.2^a^	4.524	0.045
**VH to CD ratio**	7.472	8.269	9.426	8.564	0.547	0.122

1 Values are the least squares means representing six observations.

2 VH to CD ratio, villus height to crypt depth

3 CON, control diet; BS, control + Sporezyme-forte (*Bacillus subtilis* 5.0 × 10^7^ cfu/kg diet); BL, control + Syner-win (*Bacillus licheniformis* 1.0 × 10^8^ cfu/kg diet); BSL, control + Syner-win Plus (*Bacillus subtilis* and *Bacillus licheniformis* each 1.0 × 10^8^ cfu/kg diet, total 2.0 × 10^8^ cfu/kg diet).

4 SEM, standard errors of the means.

a,b Values with different superscripts within a row differ significantly (*P *< 0.05).

### Effect on corticosterone in serum and feather samples

The corticosterone contents ranged from 206.8 to 245.8 pg/mL of serum samples, and 6.1 to 8.0 pg/mg of feather samples at 21 days of age, and elevated to 361.6 to 434.7 pg/mL of serum samples and 7.4 to 12.5 pg/mg of feather samples at 35 days of age. However, none of the dietary probiotics affected the corticosterone concentrations in the serum and feather samples at 21 and 35 days (*P *> 0.05). Although feather corticosterone levels tended to be lower by an average of 40.9% in the BSL vs. the control groups, no statistical difference was noted (*P *= 0.093; Table 6).

**Table 6:** Effects of dietary *Bacillus*-based commercial probiotics on concentrations of corticosterone in serum and feather samples of broiler chickens.^1^

Item	Treatments				SEM^3^	*P*-value
	CON^2^	BS	BL	BSL		
**Day 21**						
**Serum, pg/mL**	206.8	238.0	214.5	245.8	24.34	0.698
**Feather, pg/mg**	7.633	6.098	7.965	7.939	1.038	0.574
**Day 35**						
**Serum, pg/mL**	432.3	434.7	374.8	361.6	30.38	0.314
**Feather, pg/mg**	12.48	11.86	8.193	7.378	1.510	0.093

1 Values are the least squares means representing six observations.

2 CON, control diet; BS, control + Sporezyme-forte (*Bacillus subtilis* 5.0 × 10^7^ cfu/kg diet); BL, control + Syner-win (*Bacillus licheniformis* 1.0 × 10^8^ cfu/kg diet); BSL, control + Syner-win Plus (*Bacillus subtilis* and *Bacillus licheniformis* each 1.0 × 10^8^ cfu/kg diet, total 2.0 × 10^8^ cfu/kg diet).

3 SEM, standard errors of the means.

### Effect on antioxidant markers in serum samples

None of the dietary treatments affected antioxidant and oxidative stress markers at 21 and 35 days (*P *> 0.05). The concentration of TAC, a non-enzymatic antioxidant marker, in the serum samples at 35 days was the lowest in the BSL group, but the difference was not statistically significant compared with the control group (*P *> 0.05; Table 7).

**Table 7:** Effects of dietary *Bacillus*-based commercial probiotics on antioxidant and oxidative stress markers in serum samples of broiler chickens.^1^

Item^2^	Treatments				SEM	*P*-value
	CON^3^	BS	BL	BSL		
**Day 21**						
**GPX activity, U/L**	177.1	191.9	155.0	199.7	24.85	0.618
**SOD activity, U/L**	0.212	0.215	0.213	0.216	0.002	0.666
**CAT, U/mL**	7.337	7.248	7.653	7.784	1.369	0.993
**TAC, mM**	1.187	1.181	1.092	1.315	0.089	0.393
**MDA, µM**	7.053	7.869	6.912	9.229	1.034	0.445
**Day 35**						
**GPX activity, U/L**	476.4	405.9	352.2	426.8	56.71	0.499
**SOD activity, U/L**	0.216	0.215	0.216	0.214	0.002	0.908
**CAT, U/mL**	4.925	4.196	6.131	7.208	1.229	0.425
**TAC, mM**	1.118	1.034	1.102	0.919	0.055	0.086
**MDA, µM**	10.29	8.278	11.05	9.018	1.201	0.423

1 Values are the least squares means representing six observations.

2 GPX, glutathione peroxidase; SOD, superoxide dismutase; CAT, catalase; TAC, total antioxidant capacity; MDA, malondialdehyde.

3 CON, control diet; BS, control + Sporezyme-forte (*Bacillus subtilis* 5.0 × 10^7^ cfu/kg diet); BL, control + Syner-win (*Bacillus licheniformis* 1.0 × 10^8^ cfu/kg diet); BSL, control + Syner-win Plus (*Bacillus subtilis* and *Bacillus licheniformis* each 1.0 × 10^8^ cfu/kg diet, total 2.0 × 10^8^ cfu/kg diet).

4 SEM, standard errors of the means.

### Effect on antioxidant markers and sIgA in ileal mucosa samples

On day 21, broilers fed diets containing BS and BSL exhibited lower GPX activity by 36.34% and 38.06%, respectively in the ileal mucosa samples compared to the control group (*P *< 0.05). However, dietary *Bacillus*-based probiotic preparations did not affect SOD, CAT, TAC, and MDA on day 21 and GPX, SOD, CAT, TAC, and MDA on day 35 (*P *> 0.05). In addition, the concentration of sIgA, which plays a role in mucosal immunity, was not altered by the dietary probiotics at 21 or 35 days of age (*P *> 0.05; Table 8).

**Table 8:** Effects of dietary *Bacillus*-based commercial probiotics on antioxidant markers and secretory immunoglobulin A (sIgA) in ileal mucosa samples of broiler chickens.^1^

Item^2^	Treatments				SEM^4^	*P*-value
	CON^3^	BS	BL	BSL		
**Day 21**						
**GPX activity, U/mg of protein**	54.23^a^	34.52^b^	36.19^a,b^	33.59^b^	5.417	0.034
**SOD activity, U/g of protein**	43.00	33.58	35.24	30.27	4.425	0.366
**CAT, U/mg of protein**	2.317	2.277	3.069	2.721	0.411	0.479
**TAC, mM/mg of protein**	293.4	229.4	263.4	238.0	22.55	0.255
**MDA, nM/mg of protein**	1.167	1.018	1.392	1.154	0.169	0.487
**sIgA, µg/mg of protein**	3.257	2.611	2.376	2.448	0.605	0.737
**Day 35**						
**GPX activity, U/mg of protein**	22.80	38.82	23.90	33.24	6.484	0.278
**SOD activity, U/mg of protein**	28.08	20.95	29.00	26.95	4.970	0.709
**CAT, U/mg of protein**	0.944	2.307	2.011	1.210	0.526	0.299
**TAC, mM/mg of protein**	379.1	369.5	463.8	419.0	46.90	0.497
**MDA, nM/mg of protein**	2.140	1.959	1.543	2.065	0.203	0.253
**sIgA, µg/mg of protein**	4.726	7.406	4.699	5.607	1.226	0.519

1 Values are the least squares means representing six observations.

2 GPX, glutathione peroxidase; SOD, superoxide dismutase; CAT, catalase; TAC, total antioxidant capacity; MDA, malondialdehyde.

3 CON, control diet; BS, control + Sporezyme-forte (*Bacillus subtilis* 5.0 × 10^7^ cfu/kg diet); BL, control + Syner-win (*Bacillus licheniformis* 1.0 × 10^8^ cfu/kg diet); BSL, control + Syner-win Plus (*Bacillus subtilis* and *Bacillus licheniformis* each 1.0 × 10^8^ cfu/kg diet, total 2.0 × 10^8^ cfu/kg diet).

4 SEM, standard errors of the means.

a,b Values with different superscripts within a row differ significantly (*P *< 0.05).

### Effect on relative concentrations of cecal short-chain fatty acids

At all ages, acetate, butyrate, and propionate were the major short-chain fatty acids and branched-chain fatty acids (isobutyrate, isovalerate, and valerate) that were maintained at less than 3.0% in the cecal digesta. Nonetheless, none of the SCFAs assayed at 21 or 35 days were altered by the dietary probiotics (*P *> 0.05; Table 9).

**Table 9:** Effects of dietary *Bacillus*-based commercial probiotics on the relative concentrations (% of total) of cecal short-chain fatty acids in broiler chickens.^1^

Item^2^	Treatments				SEM^4^	*P*-value
	CON^3^	BS	BL	BSL		
**Day 21**						
**Acetate**	76.85	74.89	77.21	78.22	2.554	0.882
**Propionate**	4.504	5.902	5.432	6.055	0.669	0.596
**Isobutyrate**	0.795	0.626	0.933	0.957	0.159	0.566
**Butyrate**	16.70	16.97	14.56	12.70	1.813	0.577
**Isovalerate**	0.543	0.609	0.856	0.818	0.198	0.752
**Valerate**	0.610	1.002	1.006	1.251	0.148	0.235
**SCFA**	98.05	97.76	97.21	96.97	0.458	0.587
**BCFA**	1.948	2.237	2.794	3.026	0.458	0.587
**Day 35**						
**Acetate**	64.30	69.92	69.51	71.21	2.381	0.237
**Propionate**	10.04	7.299	8.695	7.317	0.850	0.117
**Isobutyrate**	0.827	1.129	1.180	0.845	0.142	0.266
**Butyrate**	22.51	18.89	17.72	18.32	2.140	0.451
**Isovalerate**	1.056	1.266	1.277	0.892	0.195	0.540
**Valerate**	1.272	1.499	1.614	1.417	0.136	0.446
**SCFA**	96.85	96.11	95.93	96.85	0.442	0.418
**BCFA**	3.155	3.893	4.070	3.153	0.442	0.418

1 Values are the least squares means representing six observations.

2 SCFA, short-chain fatty acid (acetate + propionate + butyrate); BCFA, branched-chain fatty acid (isobutyrate + valerate + isovalerate).

3 CON, control diet; BS, control + Sporezyme-forte (*Bacillus subtilis* 5.0 × 10^7^ cfu/kg diet); BL, control + Syner-win (*Bacillus licheniformis* 1.0 × 10^8^ cfu/kg diet); BSL, control + Syner-win Plus (*Bacillus subtilis* and *Bacillus licheniformis* each 1.0 × 10^8^ cfu/kg diet, total 2.0 × 10^8^ cfu/kg diet).

4 SEM, standard errors of the means.

### Effect on serum biochemical parameters

Serum GOT at 21 d was lowered by 24.04% and 22.27% in birds fed the BL and BSL diets, respectively, compared with the control group (*P *< 0.05; Table 10). Serum GPT concentration tended to be lower by 9.39% in the BL group than in the control group (*P *= 0.071). Other serum biochemical parameters (e.g. cholesterol, triglyceride, total protein, albumin, globulin, glucose, uric acid, creatinine, or nitric oxide) at 21 and 35 days were not altered by the dietary probiotic treatments (*P *> 0.05).

**Table 10:** Effects of dietary *Bacillus* probiotic-based commercial products on serum biochemical parameters in broiler chickens.^1^

Item^2^	Treatments				SEM^4^	*P*-value
	CON^3^	BS	BL	BSL		
**Day 21**						
**TCHO, mg/dL**	101.5	92.25	88.67	95.00	4.552	0.378
**Triglyceride, mg/dL**	55.40	69.50	56.00	57.75	7.848	0.624
**HDL-CHO, mg/dL**	77.80	77.20	63.40	72.00	6.752	0.429
**Total protein, g/L**	3.167	2.800	3.020	2.767	0.215	0.215
**Albumin, g/L**	0.767	0.720	0.700	0.767	0.066	0.855
**Globulin, g/L**	2.520	2.080	2.117	2.000	0.252	0.544
**Glucose, mg/dL**	284.3	264.8	269.5	310.2	16.98	0.347
**GPT, IU/L**	28.33	28.75	25.67	28.20	0.806	0.071
**GOT, IU/L**	186.3^a^	155.0^a,b^	141.5^b^	144.8^b^	10.95	0.042
**Uric acid, mg/dL**	9.580	8.220	9.040	10.85	0.929	0.235
**Creatinine, mg/dL**	1.867	1.675	1.620	1.367	0.124	0.074
**Nitric oxide, µM**	22.06	23.02	22.87	27.36	3.820	0.722
**Day 35**						
**TCHO, mg/dL**	114.0	105.0	101.3	90.80	7.947	0.295
**Triglyceride, mg/dL**	43.60	48.80	29.60	24.67	9.126	0.313
**HDL-CHO, mg/dL**	87.00	58.50	55.80	57.00	12.18	0.266
**Total protein, mg/dL**	1.583	1.633	1.360	1.400	0.172	0.638
**Albumin, g/dL**	0.600	0.617	0.583	0.517	0.113	0.929
**Globulin, g/L**	0.880	1.017	0.880	0.883	0.100	0.662
**Glucose, mg/dL**	196.7	195.0	212.3	182.2	18.35	0.718
**GPT, IU/L**	28.00	26.50	24.67	25.40	1.108	0.204
**GOT, IU/L**	145.0	184.2	135.8	214.2	24.08	0.142
**Uric acid, mg/dL**	5.460	6.250	5.280	4.867	0.699	0.666
**Creatinine, mg/dL**	0.883	0.383	0.300	0.317	0.168	0.076
**Nitric oxide, µM**	23.94	39.50	25.81	23.17	4.811	0.096

1 Values are the least squares means representing six observations.

2 TCHO, total cholesterol; HDL-CHO, high-density lipoprotein cholesterol; GOT, glutamic oxaloacetic transaminase; GPT, glutamic pyruvic transaminase.

3 CON, control diet; BS, control + Sporezyme-forte (*Bacillus subtilis* 5.0 × 10^7^ cfu/kg diet); BL, control + Syner-win (*Bacillus licheniformis* 1.0 × 10^8^ cfu/kg diet); BSL, control + Syner-win Plus (*Bacillus subtilis* and *Bacillus licheniformis* each 1.0 × 10^8^ cfu/kg diet, total 2.0 × 10^8^ cfu/kg diet).

4 SEM, standard errors of the means.

a,b Values with different superscripts within a row differ significantly (*P *< 0.05).

### Effect on ileal nutrient digestibility

At 35 days, dietary probiotics did not affect the apparent ileal digestibility of dry matter, ether extracts, and neutral detergent fiber (*P *> 0.05; Table 11). However, dietary BSL, but not BS or BL, significantly lowered the apparent ileal digestibility of crude protein by 6.58% and crude ash by 21.85% compared to the control (*P *< 0.05).

**Table 11:** Effects of dietary *Bacillus* probiotic-based commercial products on ileal digestibility of nutrients in broiler chickens.^1^

Item^2^	Treatments				SEM^4^	*P*-value
	CON^3^	BS	BL	BSL		
**Dry matter, %**	93.62	93.84	93.63	93.33	0.241	0.527
**Ether extracts, %**	76.26	70.82	77.64	76.78	2.817	0.348
**Crude protein, %**	81.11^a^	80.76^a^	80.08^a^	75.77^b^	0.855	0.001
**Crude ash, %**	28.42^a^	28.37^a^	30.48^a^	22.21^b^	1.253	0.001
**NDF, %**	15.11	25.42	18.01	17.38	3.638	0.207

1 Values are the least squares means representing six observations.

2 NDF, neutral detergent fiber.

3 CON, control diet; BS, control + Sporezyme-forte (*Bacillus subtilis* 5.0 × 10^7^ cfu/kg diet); BL, control + Syner-win (*Bacillus licheniformis* 1.0 × 10^8^ cfu/kg diet); BSL, control + Syner-win Plus (*Bacillus subtilis* and *Bacillus licheniformis* each 1.0 × 10^8^ cfu/kg diet, total 2.0 × 10^8^ cfu/kg diet).

4 SEM, standard errors of the means.

a,b Values with different superscripts within a row differ significantly (*P *< 0.05).

### Effect on gene expressions in the ileum

Dietary BL increased the relative expression of IL-8 gene by 2.2-fold at 21 days and of IFN-α gene by 2.1- and 2.2-fold at both 21 and 35 days compared to the control group (*P *< 0.05; Tables 12 and 13). Except for IL-8 and IFN-α genes, none of the inflammatory cytokines or inflammation-related factors were altered by dietary probiotics. For the gut barrier-related genes, the expression of CLDN1 (21 and 35 days), JAM2 (21 days), ZO1 (21 and 35 days), ZO2 (35 days) was up-regulated by 2.0- to 4.0-fold by dietary BL and BSL compared to the control group (*P *< 0.05). The expression of JAM2 at day 35 was increased by 2.5-fold in the BL diet-fed broilers compared to the control group (*P *< 0.05). The expression of MUC2 was up-regulated by dietary BL at 21 days and by all *Bacillus*-based probiotic-fed chickens at 35 days compared to the control group (*P *< 0.05). However, the relative expression of the CLDN2 and OCLDN genes was not altered by any of the *Bacillus*-added diets (*P *> 0.05). For antioxidant enzymes, the expression of SOD-1 was increased by dietary BL (*P *< 0.05), but that of GPX-1 was not altered by any of the dietary probiotics (*P *> 0.05). Finally, the expression of GLUT2 at 21 and 35 days were increased by 2.0-fold in the dietary BS, BL, and BSL groups compared to the control group (*P *< 0.05).

**Table 12:** Effects of dietary *Bacillus* probiotic-based commercial products on gene expressions on day 21 in the ileum of broiler chickens.^1^

Item^2^	Treatments				SEM^4^	*P*-value
	CON^3^	BS	BL	BSL		
**Immune-related genes**						
**IL-1β**	1.032	1.184	1.041	1.075	0.186	0.930
**IL-2**	1.031	1.099	2.190	2.117	0.348	0.067
**IL-4**	1.050	0.665	2.154	1.775	0.546	0.235
**IL-6**	1.006	0.969	2.801	2.314	0.781	0.257
**IL-8**	0.908^b^	1.012^a,b^	2.004^a^	1.718^a,b^	0.244	0.020
**IL-10**	1.015	1.069	1.728	1.897	0.355	0.205
**IL-12**	1.030	0.883	2.004	1.849	0.423	0.175
**IL-18**	1.058	0.713	1.215	1.106	0.151	0.143
**IFN-α**	1.080^b^	1.259^a,b^	2.297^a^	2.154^a,b^	0.299	0.016
**TNF-α**	1.005	1.224	1.474	1.296	0.180	0.313
**TNFSF15**	1.083	1.098	1.476	1.446	0.221	0.421
**NF-kB**	1.082	1.143	1.370	1.195	0.209	0.774
**TLR4**	1.008	0.828	1.486	1.127	0.213	0.203
**Gut barrier function-related genes**						
**CLDN1**	1.120^b^	1.091^b^	4.215^a^	3.637^a^	0.319	<0.001
**CLDN2**	1.268	2.359	2.190	2.662	0.373	0.089
**OCLDN**	1.073	0.731	0.880	0.940	0.240	0.768
**JAM2**	1.084^b^	0.911^b^	2.696^a^	2.075^a^	0.227	<0.001
**ZO1**	1.052^c^	1.530^b,c^	2.488^a,b^	2.609^a^	0.232	0.001
**ZO2**	1.140	1.778	2.000	1.930	0.344	0.257
**MUC2**	1.140^b^	1.351^b^	3.021^a^	2.354^a,b^	0.321	0.002
**Antioxidant genes**						
**SOD-1**	1.063^b^	1.083^b^	2.821^a^	2.572^a,b^	0.410	0.008
**GPx-1**	7.832	5.238	4.619	8.134	2.844	0.749
**Nutrient transporter gene**						
**GLUT2**	1.040^b^	2.317^a^	2.337^a^	2.247^a^	0.304	0.012

1 Values are the least squares means representing six observations.

2 IL-1β, interleukin-1 beta; IL-2, interleukin-2; IL-4, interleukin-4; IL-6, interleukin-6; IL-8, interleukin-8; IL-10, interleukin-10; IL-12, interleukin-12; IL-18, interleukin-18; IFN-α, interferon-alpha; TNF-α, tumor necrosis factor-alpha; TNFSF15, tumor necrosis factor superfamily member 15; NF-κB, nuclear factor kappa-light-chain-enhancer of activated B cells; TLR4, toll-like receptor 4; CLDN1, claudin-1; CLDN2, claudin-2; OCLDN, occludin; JAM2, junctional adhesion molecule 2; ZO-1, zonula occludens-1; ZO-2, zonula occludens-2; MUC2, mucin 2; SOD1, superoxide dismutase 1; GPx-1, glutathione peroxidase 1; GLUT2, glucose transporter 2.

3 CON, control diet; BS, control + Sporezyme-forte (*Bacillus subtilis* 5.0 × 10^7^ cfu/kg diet); BL, control + Syner-win (*Bacillus licheniformis* 1.0 × 10^8^ cfu/kg diet); BSL, control + Syner-win Plus (*Bacillus subtilis* and *Bacillus licheniformis* each 1.0 × 10^8^ cfu/kg diet, total 2.0 × 10^8^ cfu/kg diet).

4 SEM, standard errors of the means.

a,b,c Values with different superscripts within a row differ significantly (*P *< 0.05).

**Table 13:** Effects of dietary *Bacillus* probiotic-based commercial products on gene expressions on day 35 in the ileum of broiler chickens.^1^

Item^2^	Treatments				SEM^4^	*P*-value
	CON^3^	BS	BL	BSL		
**Immune-related genes**						
**IL-1β**	1.064	0.950	1.087	0.904	0.165	0.871
**IL-2**	1.056	1.211	1.673	1.095	0.265	0.378
**IL-4**	1.145	0.903	1.346	0.611	0.230	0.244
**IL-6**	1.077	0.849	0.991	0.549	0.196	0.384
**IL-8**	1.029	1.125	1.642	1.791	0.195	0.053
**IL-10**	1.153	0.807	1.011	0.775	0.228	0.671
**IL-12**	1.076	0.854	1.312	0.708	0.310	0.618
**IL-18**	1.156	1.216	0.956	1.521	0.272	0.635
**IFN-α**	1.168^b^	1.418^a,b^	2.617^a^	2.466^a,b^	0.314	0.012
**TNF-α**	1.053	1.126	1.277	1.273	0.131	0.591
**TNFSF15**	1.032	1.270	1.397	1.178	0.131	0.290
**NF-kB**	1.013	1.138	0.935	0.938	0.119	0.665
**TLR4**	1.076	0.911	1.302	0.781	0.266	0.620
**Gut barrier function-related genes**						
**CLDN1**	1.112^b^	0.670^b^	4.332^a^	3.283^a^	0.244	<0.001
**CLDN2**	1.047	1.472	1.740	1.964	0.316	0.290
**OCLDN**	1.060	1.711	1.625	1.835	0.248	0.199
**JAM2**	1.059^b^	1.148^b^	2.628^a^	2.020^a,b^	0.253	0.001
**ZO1**	1.154^b^	1.948^a,b^	2.592^a^	3.078^a^	0.286	0.002
**ZO2**	1.303^b^	2.254^a,b^	3.296^a^	3.344^a^	0.399	0.008
**MUC2**	1.256^b^	3.894^a^	3.718^a^	4.572^a^	0.352	<0.001
**Antioxidant genes**						
**SOD-1**	1.092^b,c^	0.883^c^	2.260^a^	2.088^a,b^	0.247	0.003
**GPx-1**	2.965	5.239	4.127	4.023	1.233	0.612
**Nutrient transporter gene**						
**GLUT2**	1.022^b^	2.146^a^	2.420^a^	2.718^a^	0.224	0.001

1 Values are the least squares means representing six observations.

2 IL-1β, interleukin-1 beta; IL-2, interleukin-2; IL-4, interleukin-4; IL-6, interleukin-6; IL-8, interleukin-8; IL-10, interleukin-10; IL-12, interleukin-12; IL-18, interleukin-18; IFN-α, interferon-alpha; TNF-α, tumor necrosis factor-alpha; TNFSF15, tumor necrosis factor superfamily member 15; NF-κB, nuclear factor kappa-light-chain-enhancer of activated B cells; TLR4, toll-like receptor 4; CLDN1, claudin-1; CLDN2, claudin-2; OCLDN, occludin; JAM2, junctional adhesion molecule 2; ZO-1, zonula occludens-1; ZO-2, zonula occludens-2; MUC2, mucin 2; SOD1, superoxide dismutase 1; GPx-1, glutathione peroxidase 1; GLUT2, glucose transporter 2.

3 CON, control diet; BS, control + Sporezyme-forte (*Bacillus subtilis* 5.0 × 10^7^ cfu/kg diet); BL, control + Syner-win (*Bacillus licheniformis* 1.0 × 10^8^ cfu/kg diet); BSL, control + Syner-win Plus (*Bacillus subtilis* and *Bacillus licheniformis* each 1.0 × 10^8^ cfu/kg diet, total 2.0 × 10^8^ cfu/kg diet).

4 SEM, standard errors of the means.

a,b,c Values with different superscripts within a row differ significantly (*P *< 0.05).

## Discussion

This study was conducted to investigate the effects of *Bacillus*-based commercial probiotic preparations on the performance, meat and tibia quality, nutrient digestibility, antioxidant parameters, gut barrier function, and local inflammation-immunity in broiler chickens. Our results demonstrated that although body weight and body weight gain were not affected by any of the dietary probiotics, dietary BS and BSL improved the feed conversion ratio compared to the control diet-fed broilers. A decrease in crypt depth or an increase in the villus height: crypt depth ratio at 21 days was noted in BSL-supplemented diet-fed broilers compared to the control group. However, dietary BSL lowered the ileal digestibility of crude protein and crude ash compared with the control group. Dietary BS and BSL decreased the antioxidant enzyme GPX in the ileal mucosa, whereas serum GOT levels were decreased by dietary BL and BSL. In the ileum, dietary BL increased the levels of IL-8 and IFN-α genes, while BL or BSL increased the levels of genes encoding gut barrier proteins (CLDN1, JAM2, ZO1, ZO2, MUC2) compared to the control group. Dietary BL increased the expression of SOD-1 gene in the ileum but did not alter SOD levels in the serum and ileal mucosa samples. Finally, the relative expression of GLUT2 gene was increased by dietary BS, BL, and BSL. Taken together, these results indicated that dietary *Bacillus*-based probiotic preparations, especially multi-species products, improve gut health and nutrient utilization and modulate a diverse range of barrier-, nutrient transport-, antioxidant-, and immune-related parameters in the gut of broiler chickens. It should be, however, kept in mind that the probiotics used in this study are of commercially available *Bacillus*-based products but have different CFU levels per the product. It cannot be excluded that the observed differences between dietary treatments might reflect differences in probiotic dosages rather than species-specific effects. In this regard, the present findings should be interpreted as the overall effects of the specific commercial products used.

In this study, dietary BSL and BS improved the feed conversion ratio, although dietary BSL tended to increase feed intake compared with the control group. Thus, the BSL-mediated improvement in the feed conversion ratio is likely to be associated with *B. subtilis*, consistent with earlier studies reported by Opalinski et al. (2007) and Sen et al. (2012). In addition, a probiotic mixture of *B. subtilis* and *B. licheniformis* has been reported to improve the growth performance of broiler chickens ( Yang et al. 2021; Biswas et al. 2023). The improvement in feed conversion ratio following *Bacillus*-based probiotic supplementation may be attributed to modulation of the gut microbial profile (Li et al. 2016) or to enhanced intestinal barrier function, as demonstrated in the present study.

Several physicochemical properties, including pH, meat color, and cooking loss, are vital indicators of meat quality (Tang et al. 2021). Among these, meat pH affects various quality attributes, such as tenderness, water-holding capacity, color, juiciness, and shelf life (Mir et al. 2017). In broilers, optimal pH values are approximately 6.2–6.5 at 15 min postmortem and 5.8 at 24 h (Beňo et al. 2021). Meat with pH values below 5.7 is considered acidic, while that with values above 6.1 is associated with undesirable dark, firm, and dry conditions characterized by poor deliciousness (Beauclercq et al. 2022). Our study revealed that, although the breast meat with a pH from 5.85 to 5.99 was in the optimal range, meat from the dietary BS group exhibited the lowest pH, consistent with Hussein et al. (2020) and Mohammed et al. (2024), presumably because of the *B. subtilis*-altered rate of postmortem glycolytic processes in the muscle tissue.

In the present study, dietary BSL (a multi-species probiotic containing *B. subtilis* and *B. licheniformis*) led to reduced crypt depth and increased the villus height: crypt depth ratio in broilers on day 21, indicating improved intestinal morphology. A higher villus height: crypt depth ratio reflects functional efficiency of the intestinal mucosa (Jha and Mishra 2021) or greater absorptive surface (Yamauchi 2002). The enhancements in the intestinal structure noted in this study are consistent with previous reports (Musa et al. 2019; Vimon et al. 2020; Cai et al. 2024).

Free radicals can damage cell membrane lipids and produce malondialdehyde, thereby accelerating lipid peroxidation and aggravating oxidative stress (Ayala et al. 2014). Among the antioxidant and oxidative indicators, the GPX activity in the ileal mucosa at 21 days was decreased by dietary BS and BSL. GPX possesses both antioxidant and anti-inflammatory functions and is highly expressed along the crypt–villus axis, which secretes microbicidal defensins in response to bacterial challenges (Surai et al. 2018). Nonetheless, except GPX activity in the ileal mucosa, none of the antioxidant or oxidative markers in the serum or ileal mucosa at 21 and 35 days were altered by any of the dietary *Bacillus*-based probiotics. Thus, an explanation for how dietary BS and BSL lowered ileal GPX activity is not readily available at this stage.

GOT and GPT serve as biomarkers of hepatocellular damage and muscle injury (Lin et al. 2010). In this study, both BL and BSL groups showed lower GOT levels in the serum samples at 21 days, suggesting reduced liver oxidative stress or improved hepatocellular integrity. Consistent with our study, lower serum GOT levels were noted in broiler chickens fed *B. subtilis* (Mushtaq et al. 2024), *Lactobacillus paracasei* LK01 (Liu et al. 2024), and *B. licheniformis* (Elleithy et al. 2023). However, caution may be needed to interpret the *Bacillus*-led increase in liver health as manifested by a decrease in the GOT levels, as all values noted in this study fall within the physiological levels of serum GOT in broiler chickens ranging from 1.4 to 5.7 μkat/L (equivalent to 82-335 IU/L) (Zálešáková et al. 2025).

Contrary to our expectations, dietary BSL reduced the ileal digestibility of crude protein and crude ash compared with the control group. At this stage, a clear explanation for the BSL-mediated decrease in nutrient digestibility (crude protein and crude ash) was not readily available as a feed conversion ratio, and ileal morphology was improved by dietary BSL. Earlier studies on probiotics showed that dietary *Saccharomyces cerevisiae* (Khalid et al. 2021) or multi-species probiotics at 1 × 10^10^ cfu/kg of diet (Mountzouris et al. 2010) decreased the ileal or total tract digestibility of crude protein in broiler chickens. Notably, the meat yield and tibia breaking strength assayed at 35 days were not altered by dietary probiotics.

The innate immune response is a complex network of defense mechanisms that protect the host against pathogenic microorganisms and environmental challenges (Kogut et al. 2020). Interleukin-8 (IL-8) recruits immune cells to the site of infection by binding to the CXCR1 receptor on its surface, thereby contributing to immune activation and cellular defense (Yu et al. 2020). Interferon-α (IFN-α), produced by most eukaryotic cells in response to external stimuli, represents a key component of the early innate immune defense (Jiang et al. 2011). In the present study, chickens receiving a diet supplemented with *B. licheniformis* showed up-regulated expression of IL-8 and IFN-α genes, indicating a modulation of innate immune-related gene expression at the transcriptional level. However, these results of altered gene expression should be interpreted with caution as we did not measure them at the protein level. Therefore, although *B. licheniformis* may influence intestinal immune signaling, additional challenge-based or functional assays are needed to determine whether these transcriptional changes translate into functional improvements in host defense. Nonetheless, these findings align with previous studies reporting that probiotics induce upregulation of intestinal IFN-α (Xu et al. 2017; Zhu et al. 2024) and IL-8 (Zhang et al. 2024) expression in chickens.

The beneficial role of probiotics in maintaining intestinal barrier integrity by regulating tight junctions has been well documented in poultry (Chang et al. 2020; Wu et al. 2022). Tight junctions are multiprotein complexes located in the apical region of intestinal epithelial cells. They mainly comprise claudins (CLDN), zonula occludens (ZO), and junctional adhesion molecules (JAM), which together regulate paracellular permeability and protect the host from luminal antigens and pathogens (Patra and Kar 2021; Wickramasuriya et al. 2021). In the present study, dietary BL (*B. licheniformis*) or BSL (*B. subtilis* and *B. licheniformis*) enhanced the expression of genes encoding CLDN-1, JAM-2, ZO-1, and ZO-2 in the ileum. Consistent with our observations, previous studies have reported probiotic-induced upregulation of CLDN-1 (Wang et al. 2017; Zhao et al. 2022), JAM-2 (Chaudhari et al. 2020; Park et al. 2020), ZO-1 (Musa et al. 2019; Pan et al. 2022), and ZO-2 (Nii et al. 2020; Zhao et al. 2022) in chickens. MUC-2 is a secretory gel-forming mucin that serves as a crucial biological barrier protecting the intestinal epithelium from pathogens and mechanical damage (Jiang et al. 2013). In the present study, all *Bacillus*-based probiotics exhibited higher MUC-2 mRNA expression in the ileum on day 35. In contrast, a significant increase on day 21 was observed only in birds fed dietary BL (*B. licheniformis*), indicating enhanced mucin production, which is consistent with previous findings (Aliakbarpour et al. 2012; Bilal et al. 2021). However, as functional assays assessing gut barrier function including in vivo and ex vivo intestinal permeability or bacterial translocation were not measured, the *Bacillus*-mediated up-regulation of gut barrier genes noted in this study should be interpreted as suggestive of a potential enhancement of gut barrier function.

Superoxide dismutase is a crucial antioxidant enzyme that protects cells from oxidative stress by neutralizing reactive oxygen species (ROS) such as superoxide anions and hydrogen peroxide (Zheng et al. 2023). Our study indicated that dietary BL (*B. licheniformis*) increased the expression of SOD-1 gene in the ileum of broiler chickens at 21 and 35 days. This suggests that *B. licheniformis* enhances the antioxidant defense mechanisms by stimulating SOD activity. These results are similar to those of previous studies, demonstrating that probiotic strains can upregulate SOD gene expression in various chicken organ models (Memon et al. 2021; Chang and Yu 2022; Soltan et al. 2024). However, the increased SOD mRNA levels observed in our study were not accompanied by corresponding changes in enzyme activity in serum and ileal mucosa samples. This discrepancy can be explained by the complex regulation of gene expression, which occurs not only at the transcriptional level, but also through post-transcriptional mechanisms, including mRNA stability, translation efficiency, and post-translational modifications (Miao and St. Clair 2009). Consequently, elevated mRNA levels may not always translate into a proportional increase in functional enzyme activity. *B. licheniformis* may stimulate the upregulation of antioxidant genes in response to oxidative stress. Similar observations have been reported for fructooligosaccharides (Yan et al. 2024) and yeast hydrolysate from *Saccharomyces cerevisiae* (Wang et al. 2022a), which also upregulate SOD gene expression without corresponding changes in enzyme activity in various chicken tissues.

The glucose transporter GLUT2 is crucial for glucose absorption from the intestine. It facilitates the passive transport of glucose from the intestinal cells into the blood (Röder et al. 2014; Wang et al. 2022b). Upregulated genes encoding nutrient transporters can increase their expression in intestinal cells, thereby enhancing nutrient absorption from the intestinal lumen into the bloodstream (Al-Khalaifah et al. 2020). Previous studies have reported that probiotics based on dietary *Bacillus* spp. enhance intestinal glucose absorption in poultry (Han et al. 2023; Wang et al. 2023; Elbaz et al. 2024). Consistent with these studies, our study revealed that all *Bacillus*-based probiotics significantly upregulated ileal GLUT2 mRNA expression in broiler chickens at 21 and 35 days of age. The elevated expression of GLUT2 suggests that *Bacillus* supplementation may facilitate efficient glucose transport from enterocytes into circulation, thereby promoting better energy utilization and growth performance.

## Conclusion

In conclusion, three Bacillus-based commercial probiotics, single-strain or multi-species, comprising *B. subtilis* or *B. Licheniformis*, were tested as feed additives for their effects on growth performance, meat and tibia traits, nutrient digestibility, and various parameters of gut health, antioxidants, and innate immunity. It revealed that dietary BS (*B. subtilis*) improved the feed conversion ratio while dietary BL (*B. licheniformis*) increased the expression of cytokines (IL-8 and IFN-α), tight junction proteins (CLDN1, JAM2, ZO1, and ZO2), MUC2, antioxidant enzyme (SOD-1), and nutrient transport (GLUT-2) mRNA in the ileum. Thus, it is understood that the effects (improvement in feed conversion ratio and augmentation in genes encoding cytokine-, barrier-, antixodiant-, nutrient transport-related parameters) exerted by dietary BSL (*B. subtilis*, *B. licheniformis*) in broiler chickens can be explained by an additive effect of either BL and BS. Based on these findings, further studies are required to determine whether dietary *Bacillus*-based probiotics can augment or modulate protective immunity against enteric pathogens, including pathogenic *Escherichia coli*, *Clostridium perfringens*, *Salmonella* spp., and *Eimeria* spp., in broiler chickens.

## Abbreviations

AIA: acid-insoluble ash
AID: apparent ileal digestibility
AMEn: Nitrogen-corrected apparent metabolizable energy
BCFA: branched-chain fatty acid
BW: body weight
BWG: body weight gain
CAT: catalase
CD: crypt depth
CIE: commission internationale de l‘Éclairage
CLDN: claudin
CP: crude protein
DNA: deoxyribonucleic acid
ELISA: enzyme-linked immunosorbent assay
FCR: feed conversion ratio
FI: feed intake
GAPDH: glyceraldehyde-3-phosphate dehydrogenase
GLM: general linear model
GLUT: glucose transporter
GOT: glutamic oxaloacetic transaminase
GPT: glutamic pyruvic transaminase
GPX: glutathione peroxide
HDL-CHO: high-density lipoprotein cholesterol
IFN-α: interferon-alpha
IL: interleukin
JAM: junctional adhesion molecule
MDA: malondialdehyde
mRNA: messenger ribonucleic acid
MUC: mucin
NCBI: National Center for Biotechnology Information
NDF: neutral detergent fiber
NF-kB: factor nuclear factor kappa B
OCLDN: occludin
PBS: phosphate bufferd saline
PCR: polymerase chain reaction
ROS: reactive oxygen species
RT-qPCR: reverse transcription-quantitative polymerase chain reaction
SCFA: short chain fatty acid
SEM: standard errors of the means
sIgA: secretory immunoglobulin A
SOD: superoxide dismutase
TAC: total antioxidant capacity
TCHO: total cholesterol
TLR-4: toll-like receptor 4
TNFSF: tumor necrosis factor superfamily
TNF-α: tumor necrosis factor alpha
VH: villus height
ZO: zonula occludens
